# Regularities in the Evolution of Thermoelastic Martensitic Transformations during Cooling/Heating in the Free State and under Load of Titanium Nickelide Alloyed with Niobium

**DOI:** 10.3390/ma17010175

**Published:** 2023-12-28

**Authors:** Ekaterina S. Marchenko, Anatoly A. Klopotov, Gulsharat A. Baigonakova, Ilya A. Zhukov

**Affiliations:** 1 Laboratory of Superelastic Biointerfaces, National Research Tomsk State University, 36 Lenin Ave., 634045 Tomsk, Russia; 89138641814@mail.ru (E.S.M.); klopotovaa@tsuab.ru (A.A.K.); gofra930@gmail.com (I.A.Z.); 2 Institute for Problems of Chemical and Energy Technologies, Siberian Branch of the Russian Academy of Sciences, St. Socialist 1, 659322 Biysk, Russia

**Keywords:** TiNiNb, martensitic transformation, shape memory effect, structure, thermodynamics, alloying

## Abstract

This article presents the results of studies of the features of the development of thermoelastic martensitic transformations during cooling/heating in the free state and under load of Ti_50_Ni_49.7−X_Nb_X_Mo_0.3_ alloys (X = 0.5, 1.0 and 1.5 at% Nb) with shape memory effects. Using X-ray diffraction analysis, it was found that all the alloys studied at room temperature contained a multiphase mixture consisting of intermetallic compounds with the TiNi (B2, B19′), Ni_56_Ti_29_Nb_15_, and Ti_2_Ni compositions. Scanning electron microscopy was used to study the microstructure of TiNi (Nb,Mo) alloys and it was found that the distribution of fine Ni_56_Ti_29_Nb_15_ particles in the matrix depends significantly on the concentration of the alloying element. A correlation was established between changes in the structural-phase state in TiNi (Nb,Mo) alloys and the occurrence of the B2↔B19′ martensitic transition in the free state and under load. Based on physical and mechanical studies, the temperature ranges of the martensitic transformations (MT) in the free state and under load were established. Based on the thermodynamic description of the MT and the analysis of the characteristic temperatures of the MT, it was found that the MT mechanism is strongly dependent on the concentration of the alloying element.

## 1. Introduction

Titanium nickelide and alloys based on it have a set of unique physical and mechanical properties, such as the shape memory effect (SME), superelasticity. The use of this effect in alloys based on titanium nickelide is widely used in engineering and medicine [1]. The representation of SME is due to thermoelastic martensitic transformations (MT) [2]. The synthesis of alloys based on titanium nickelide with specific functional and technological properties is achieved by doping the binary NiTi alloy with a third component and by preliminary thermomechanical treatments. Ti_50−X_Ni_50−Y_Nb_X+Y_ alloys have unique properties among ternary alloys. This is because a wide hysteresis of the thermoelastic MT is observed in these alloys [3,4,5,6,7,8,9]. This property allows products and structural elements made of alloys based on TiNi(Nb) to be stored and transported over a wide range of ambient temperatures.

A large number of studies of SME manifestation features have been devoted to ternary alloys with high Nb content. There are the Ti_44_Ni_47_Nb_9_ [10,11], Ti_47_Ni_44_Nb_9_ [12,13], Ti_45.81_Ni_45.76_Nb_8.43_ [14] alloys which have been well studied. On the other hand, TiNi-based alloys with a low Nb concentration have not been extensively studied and are widely used in industry [4,5,6,7,8,9,10,15,16]. It has been found that in this Ti_47_Ni_44_Nb_9_ alloy, β-Nb particles are formed, which extend the thermal hysteresis of the MT. During plastic deformation of TiNi(Nb) alloys, the presence of more plastic β-Nb particles, in comparison with the main matrix based on the TiNi intermetallic compound, contributes to the weakening of the accumulation of energy of the elastic deformation of martensite [4,8,10,17]. One of the main features of Nb-doped TiNi alloys is the large difference in the melting temperatures of the Nb alloying element relative to the two main alloying elements Ti and Ni. This means that the formation of segregations is difficult to avoid. It can therefore be assumed that the addition of small concentrations of Nb to TiNi based alloys is the most optimal.

The aim of this work was to study the features of the development of thermoelastic martensitic transformations during cooling/heating in the free state and under the load of TiNi-based alloys with low Nb concentrations.

## 2. Materials and Methods

### 2.1. Alloy Preparation

In the induction furnace ISV-0.004 PI M1, a series of alloys Ti_50_Ni_49.7−X_Nb_X_Mo_0.3_ (X = 0.5, 1.0 and 1.5 at% Nb) was melted by remelting spongy titanium and nickel of N1 grade. The composition was determined by the charge. The weight loss of ingots during melting did not exceed 0.01%. The resulting ingots had a weight of 550 g, a length of 300 mm and a diameter of 22 mm. To study the functional properties, samples with dimensions of 50 mm in length and 1 mm × 1 mm in cross section were cut by the electro-erosive method. To carry out structural studies, 1 mm thick plates with dimensions of 10 mm × 10 mm were cut out.

### 2.2. Structure Characterization Methods

The microstructure of the alloys was studied using a Carl Zeiss Axiovert 40 MAT metallographic microscope (Oberkochen, Germany). The Thermo Scientific Axia ChemiSEM scanning electron microscope (SEM) (Waltham, MA, USA) with an energy dispersive X-ray spectrometer (EDS) was used for the quantitative elemental analysis. To study the microstructure, thin sections were prepared in a standard way. To reveal the microstructure, a solution of hydrofluoric and nitric acids was used (3 mL HF, 2 mL HNO_3_, 95 mL H_2_O).

The phase composition of the samples was studied using a DRON-4 and a Shimadzu XRD-6000 diffractometer (Kyoto, Japan) in Cokα and Cukα radiation. A monochromator was used to cut off the β-radiation. X-ray diffraction studies were carried out according to the methods described in [18]. The diffraction patterns were indexed using the PowderCell 2.4 program. The XRD analysis was carried out at the Tomsk Regional Core Shared Research Facilities Center of the National Research Tomsk State University, within grants (nos. 075-15-2021-693 and 13.RFC.21.0012) from the Ministry of Science and Higher Education of the Russian Federation.

### 2.3. The Study of the Nature of Martensitic Transformation

The study of the nature and sequence of MT was carried out by measuring the temperature dependence of the electrical resistance and by X-ray diffraction analysis. The characteristic temperatures and intervals of manifestation of the MT were determined by the potentiometric method from the temperature dependence of the electrical resistivity. To determine the MT behavior, the electrical resistivity of the samples (50 mm × 1 mm × 1 mm) was measured by the four-point-probe method in a temperature range from −180 to +180 °C [18,19]. The multiple shape memory effect test was carried out by measuring the macrostrain under a tension loading of 2 kg on an Instron testing machine with a cooling–heating chamber.

## 3. Thermodynamics of Thermoelastic Martensitic Transformations

It has been shown in [19,20] that thermoelastic MT begins when the alloy is overcooled by ΔT_overc_ relative to the chemical equilibrium temperature T_0_ of the austenite and martensite phases to a lower temperature M_S_.

At temperature T_0_, the Gibbs chemical free energy of austenite and martensite is the same. According to the Tong–Weiman formula [19,20], the temperature T is determined by the expression. It should provide a concise and accurate description of the experimental results, their interpretation and the experimental conclusions that can be drawn.

(1) $$T_{0}=\frac{M_{s+}A_{f}}{2}$$

M_S_ and A_f_ are temperatures at the beginning of the forward MT and the end of the reverse MT.

On the other hand, the relationship between the temperatures M_S_ and T_0_ can be represented as below:

(2) $$M_{s}=T_{0}-\Delta T_{\mathrm{overc}}$$

It follows from expression (2) that the temperature of direct MT depends on the magnitude of overcooling, which in turn depends on the external mechanical stresses [19,20].

In the case when direct MT occurs in alloys in the absence of external mechanical stresses, then the energy balance can be written as in [21]:

(3) $$-\Delta G_{\mathrm{ch}}^{A-M}+\Delta G_{\mathrm{nonch}}^{A-M}=0$$

Here $-\Delta G_{\mathrm{ch}}^{A-M}$ is the chemical driving force of the MT and $\Delta G_{\mathrm{nonch}}^{A-M}$ is the Gibbs free energy of a non-chemical nature. These energies are determined by the expressions in [21]:

(4) $$\Delta G_{\mathrm{ch}}^{A-M}={\Delta H}_{\mathrm{ch}}^{A-M}-T\times {\Delta S}_{\mathrm{ch}}^{A-M}$$

(5) $${\Delta G}_{\mathrm{nonch}}^{A-M}={\Delta G}_{\mathrm{rev}}^{A-M}+{\Delta G}_{\mathrm{fr}}^{A-M}$$

Here ${\Delta G}_{\mathrm{rev}}^{A-M}$ is the reversible component of the energy, which is the sum of the elastic and surface energies. These energies are accumulated in the alloy during direct MT and depend on the volume fraction of the martensite phase δ; ${\Delta G}_{\mathrm{fr}}^{A-M}$ is irreversible non-chemical free energy, which is responsible for the irreversible dissipation of energy in the MT process; ${\Delta S}_{\mathrm{ch}}^{A-M}$ is entropy of MT.

In [22], expressions were obtained for the characteristic temperatures of the MT:

(6a) $$M_{s}=T_{0}-\frac{\left|\Delta G_{\mathrm{rev}}\left.\left(0\right)\right|\right.}{\Delta S_{\mathrm{ch}}}-\frac{\left|\Delta \right.\left.G_{\mathrm{fr}}\right|}{\Delta S_{\mathrm{ch}}}$$

(6b) $$M_{f}=T_{0}-\frac{\left|\Delta G_{\mathrm{rev}}\left.\left(1\right)\right|\right.}{\Delta S_{\mathrm{ch}}}-\frac{\left|\Delta \right.\left.G_{\mathrm{fr}}\right|}{\Delta S_{\mathrm{ch}}}$$

(6c) $$A_{s}=T_{0}-\frac{\left|\Delta G_{\mathrm{rev}}\left.\left(1\right)\right|\right.}{\Delta S_{\mathrm{ch}}}+\frac{\left|\Delta \right.\left.G_{\mathrm{fr}}\right|}{\Delta S_{\mathrm{ch}}}$$

(6d) $$A_{f}=T_{0}-\frac{\left|\Delta G_{\mathrm{rev}}\left.\left(0\right)\right|\right.}{\Delta S_{\mathrm{ch}}}+\frac{\left|\Delta \right.\left.G_{\mathrm{fr}}\right|}{\Delta S_{\mathrm{ch}}}$$

Here $\Delta G_{\mathrm{rev}}(0)$ and $\Delta G_{\mathrm{rev}}(1)$ are the reversible elastic $\Delta G_{\mathrm{el}}$ and surface energies $\Delta G_{s}$, accumulated in the alloy during direct MT at the volume fraction of the martensite phase δ = 0 and δ = 1, respectively ($\Delta G_{\mathrm{rev}}=\Delta G_{\mathrm{el}}+\Delta G_{s}$). With direct MT, the entropy of transformation is $\Delta S_{\mathrm{ch}}^{A-M}<0$, and with reverse MT $\Delta S_{\mathrm{ch}}^{A-M}>0$; then in Equations (6a)–(6d) the entropy is ${\Delta S}_{\mathrm{ch}}=|{\Delta S}_{\mathrm{ch}}^{A-M}|$.

Transforming Equations (6a) and (6b), we obtain the following expressions:

$$\Delta G_{\mathrm{fr}}=\frac{{\Delta S}_{\mathrm{ch}}}{2}(A_{f}-M_{s})$$

(7) $$\left(A_{f}-M_{s}\right)=\frac{2\Delta G_{\mathrm{fr}}}{{\Delta S}_{\mathrm{ch}}}$$

(8) $$\left(A_{s}-M_{s}\right)=\frac{1}{{\Delta S}_{\mathrm{ch}}}(2\left|\Delta \right.\left.G_{\mathrm{fr}}\right|-\left|\Delta \right.\left.G_{\mathrm{rev}}\right|)$$

(9) $$\left|\Delta \right.\left.G_{\mathrm{rev}}(1)\right|=\left(M_{s}-M_{f}\right)\frac{\Delta S_{\mathrm{ch}}}{2}+(A_{f}-A_{s})\frac{\Delta S_{\mathrm{ch}}}{2}$$

From the expressions obtained (7)–(9), there are important consequences. First, energy dissipation $\Delta G_{\mathrm{fr}}$ determines the amount of thermal hysteresis $\left(A_{f}-A_{s}\right)$. Second, the value of the temperature intervals of direct $(\Delta ↑=M_{s}-M_{f}$) and reverse ($\Delta ↓=A_{f}-A_{s})$ MT is dependent in direct proportion to the reversible component of free energy. Third, the temperature difference between the beginning of the direct and the beginning of the reverse ($A_{s}-M_{s}$) MT is determined by the ratio between the reversible $\left|\Delta \right.\left.G_{\mathrm{rev}}(1)\right|$ and irreversible $\left|\Delta \right.\left.G_{\mathrm{fr}}\right|$ energies. Fourth, when the relation $A_{s}<M_{s}$ is observed, then the condition $\left|\Delta \right.\left.G_{\mathrm{fr}}\right|$ < 0.5$\left|\Delta \right.\left.G_{\mathrm{rev}}(1)\right|$ is satisfied.

The theoretical consideration of the features of a thermoelastic MT presented in this section will be used in this paper to analyze the obtained experimental data.

## 4. Results and Discussion

### 4.1. Structural and Phase States of TiNi-Based Alloys Doped with Nb

X-ray diffraction studies of TiNi-based alloys doped with Nb have shown that the alloy contains an austenitic phase with a B2 structure, a martensitic phase with a B19′ structure, and a secondary Ti_2_Ni phase. No data have been found to indicate the formation of additional niobium-based phases. The intensity of reflections from the secondary Ti_2_Ni phase is low. The estimate shows that the amount of the Ti_2_Ni phase does not exceed 5%.

**Figure 1:**
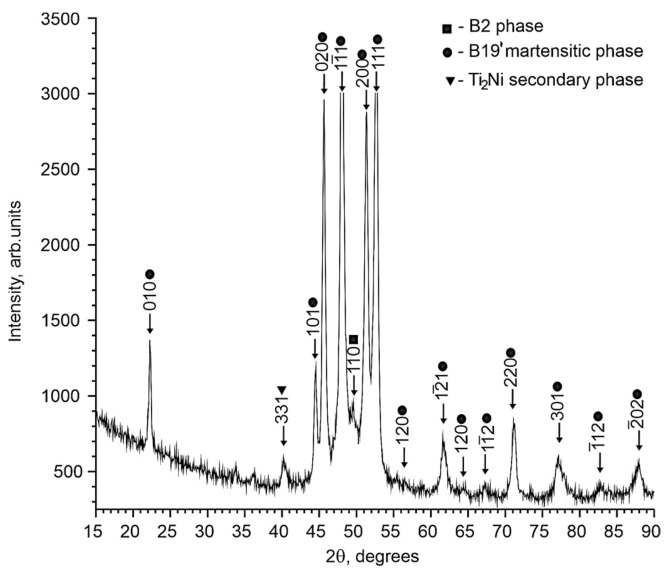
X-ray diffraction pattern of the Ti_50_Ni_49.2_Nb_0.5_Mo_0.3_ alloy sample, obtained with Cokα radiation.

**Figure 2:**
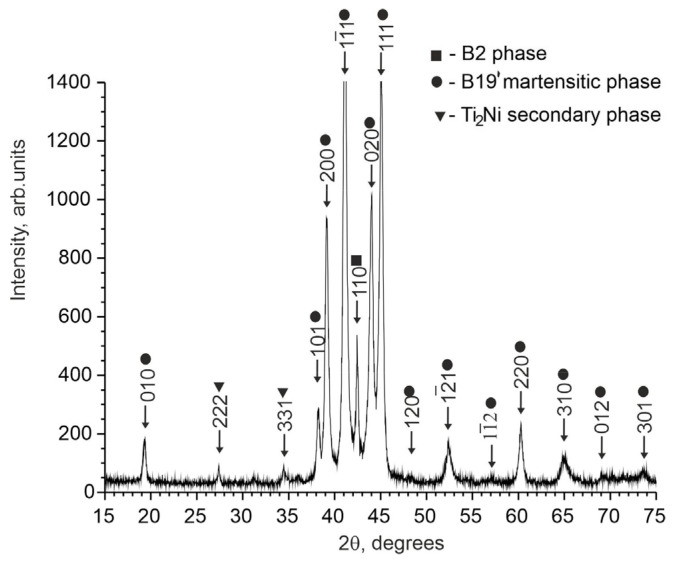
X-ray diffraction pattern of the Ti_50_Ni_48.7_Nb_1_Mo_0.3_ alloy sample obtained with Cukα radiation.

**Figure 3:**
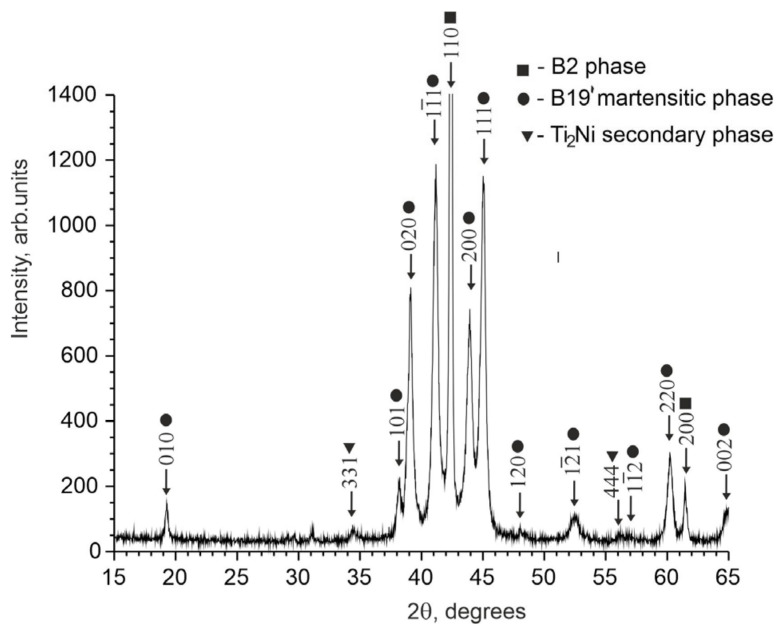
X-ray diffraction pattern of the Ti_50_Ni_48.2_Nb_1.5_Mo_0.3_ alloy sample obtained with Cukα radiation.

Analysis of the X-ray patterns of the alloys studied made it possible to establish that all the X-ray patterns contain structural reflections of the B19′ martensitic phase with high intensity (Figure 1 and Figure 2). Moreover, the ratio between the intensities of the main reflections between the austenitic and martensitic phases depends on the concentration of the alloying element Nb. Thus, in alloys with an alloying element concentration of less than 1.5 at% Nb, the intensity of the main reflection (110)B2 of the austenitic phase B2 is less than the intensity of the reflection ${\left(1\bar{1}1\right)}_{B19\prime }$ of the martensite phase B19′, i.e., ($I_{110(B2)}<I_{1\bar{1}1(B19\prime )}$. In alloys with an alloying element concentration 1.5 at% Nb, on the contrary, $I_{110(B2)}>I_{1\bar{1}1(B19\prime )}$ (Figure 1, Figure 2 and Figure 3).

**Figure 4:**
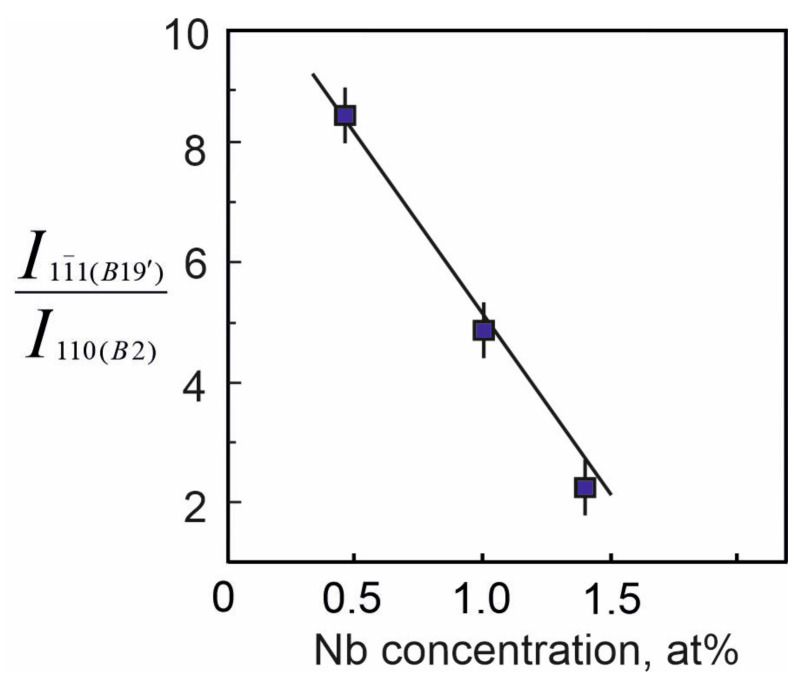
Dependence of the ratio of reflection intensities $\left(1\bar{1}1\right)$ on the martensitic phase with the B19′ structure and (110) on the austenitic phase with the B2 structure on the concentration of Nb atoms.

Figure 4 shows the dependence of the ratio of the intensities of the structural line of the martensite phase B19′ to the structural line of the austenite phase B2 on the concentration of Nb atoms in the alloys under study.

It should be noted from this dependence that as the Nb concentration increases, the ratio of the intensity of the reflection ${\left(1\bar{1}1\right)}_{B19\prime }$ to the intensity of the reflection (110)_B2_ decreases. This indicates that alloying alloys based on titanium nickelide with niobium leads to an increase in the stability of the high-temperature B2 phase at room temperature relative to the B19′ martensitic phase.

**Figure 5:**
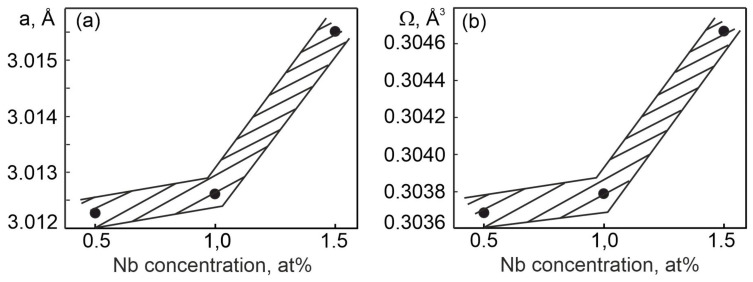
Concentration dependences of the unit cell parameter in the B2 phase (**a**) and atomic volume (**b**) in TiNi alloys doped with niobium.

The unit cell parameter of the B2 phase was determined for alloys with different contents of niobium. Based on the obtained values of the unit cell parameters, we calculated the change in the atomic volume in the B2 phase as a function of the Nb concentration (Figure 5).

It can be seen that with an increase in the concentration of niobium atoms, an increase in the lattice parameter and atomic volume of the B2 phase is observed. This indicates that some of the Nb atoms are dissolved in the B2 matrix phase. It should be noted here that a nonlinear increase in the lattice parameter is observed depending on the concentration of the alloying element. This may indicate a more significant dissolution of Nb atoms in the crystal lattice in the TiNi compound with the B2 structure, or it may also indicate the formation of a larger amount of Ti_2_Ni secondary phase particles than in other alloys with a lower concentration of the Nb alloying element.

The resulting increase in the lattice parameter demonstrates an increase in the atomic volume per ion in the crystal lattice of the B2 phase. This phenomenon correlates with the fact that the size of the atoms of the Nb alloying element is larger than the size of the atoms of the main alloy-forming elements Ti and Ni (R_Ti_ = 0.1462 nm, R_Ni_ = 0.1246 nm and R_Nb_ = 0.1468 nm). Niobium, according to microanalysis data, dissolved in the matrix phase with the B2 (B19′) structure in an amount from 0.18 to 0.49 at%.

### 4.2. Microstructure of TiNi-Based Alloys Doped with Nb

**Figure 6:**
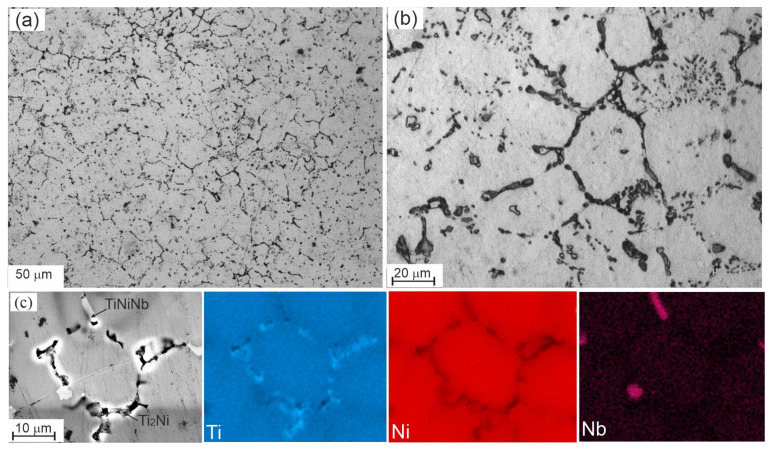
Microstructure of the Ti_50_Ni_49.2_Nb_0.5_Mo_0.3_ alloy obtained by optical (**a**,**b**) and scanning electron microscopy (**c**) with corresponding EDS maps.

**Figure 7:**
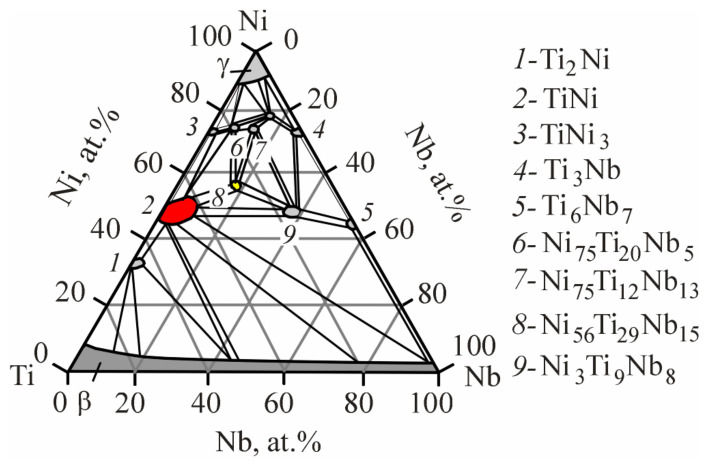
The 800 °C isothermal triangle of the Ti-Ni-Nb ternary system.

**Figure 8:**
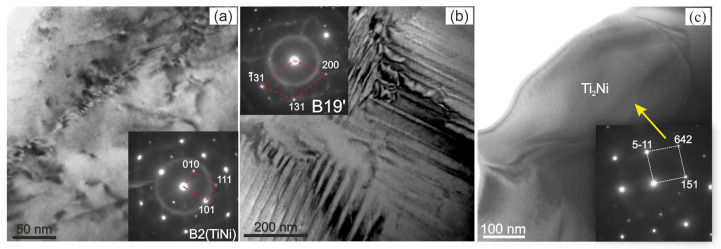
TEM image of the Ti_50_Ni_49.2_Nb_0.5_Mo_0.3_ alloy structure with corresponding diffraction patterns of B2 (**a**), B19′ (**b**) and Ti_2_Ni (**c**).

**Figure 9:**
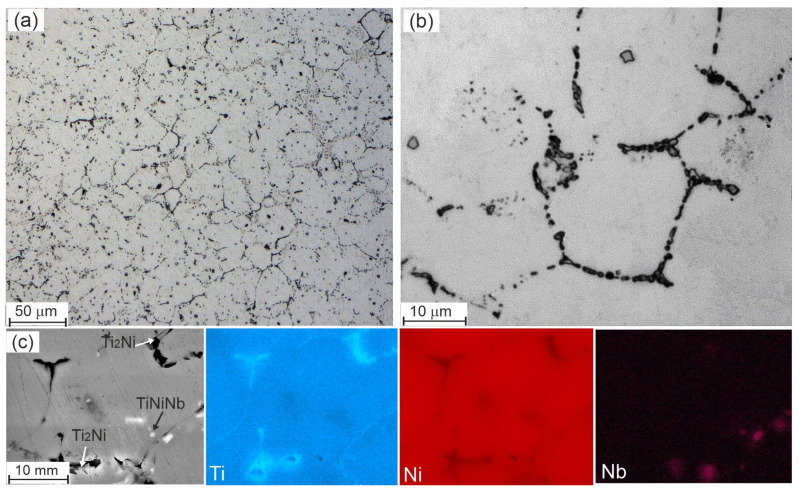
Microstructure of the Ti_50_Ni_48.7_Nb_1_Mo_0.3_ alloy obtained by optical (**a**,**b**) and scanning electron microscopes (**c**) with corresponding EDS maps.

**Figure 10:**
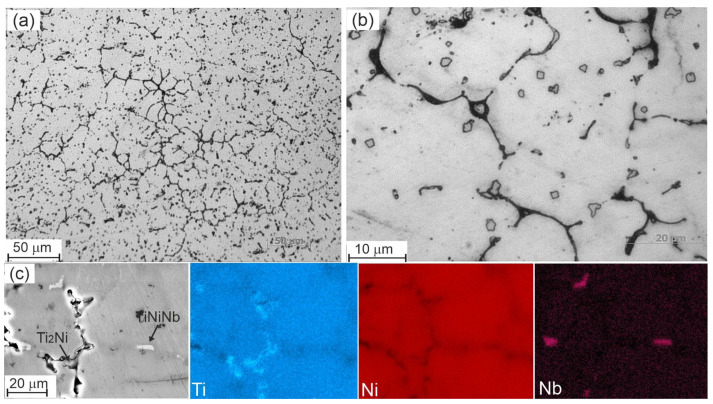
Microstructure of the Ti_50_Ni_48.2_Nb_1.5_Mo_0.3_ alloy obtained by optical (**a**,**b**) and scanning electron microscopes (**c**) with corresponding EDS maps.

Figure 6, Figure 7, Figure 8, Figure 9 and Figure 10 show micrographs of Nb-doped TiNi-based alloys. Analysis of the micrographs revealed a mixture of the solid solution melt with areas of granular crystallization and eutectics. The microstructure of each alloy is different and depends on the concentration of the alloying element. In all the alloys studied, particles of arbitrary geometry with sizes in the order of 1–2 µm were found, located both along the grain boundaries and within the grain body in the form of individual small inclusions and local accumulations (Figure 6, Figure 9 and Figure 10. Individual grain boundaries are occupied by another type of particle, Ti_2_Ni of 0.2 to 1 µm in size, formed in the form of chains (Figure 6, Figure 9 and Figure 10).

Ti_50_Ni_49.2_Nb_0.5_Mo_0.3_ alloy. Qualitative and quantitative microanalysis of the Ti_50_Ni_49,2_Nb_0.5_Mo_0,3_ alloy was carried out by scanning electron microscopy (Figure 6, Table 1). Elemental distribution maps and local quantitative microanalysis made it possible to identify the matrix based on the intermetallic compound B2—TiNi, Ti_2_Ni, and Ni_56_Ti_29_Nb_15_ phases (Figure 6a–c). The Ni_56_Ti_29_Nb_15_ phase crystallizes in the form of light particles in the grain body. According to the results of EDS microanalysis (Ni = 54–57 at%, Ti 28–31 at% and Nb = 13–16 at%) and comparison of the data with the Ti-Ni-Nb triple state diagram [23] (Figure 7), it can be assumed that this is a phase of the Ni_56_Ti_29_Nb_15_ type. The presence of a Ti_2_Ni secondary intermetallic phase along the grain boundaries of the TiNi matrix was also observed (Figure 6c). As a result of Ti redistribution, Ti_2_Ni particles are formed from the TiNi matrix phase. This leads to an increase in the nickel concentration in the matrix phase. The average grain size in the alloy of this composition is 15 μm. It was also found that the area inside the grains contains a small amount of Nb particles (up to 0.28 at%) in addition to titanium and nickel, indicating dissolution of Nb in the matrix phase.

A feature of the phase diagram of the Ti-Ni-Nb ternary system is that it reflects the features of the phase diagrams of the Ti-Ni, Ni-Nb and Ti-Nb binary systems only in limited areas adjacent to the sides and corners of the isothermal triangle [23,24]. In the Ti-Nb binary system, immediately after solidification, the components form a continuous solid solution over the entire plane in temperature-concentration coordinates, and this state extends to low temperatures. In the Ti-Ni and Ni-Nb systems, on the other hand, there are intermediate intermetallic compounds with narrow regions of homogeneity [24]. On the side of the isothermal Ti-Ni triangle at a temperature of 800 °C in the Ti-Ni-Nb ternary system, in the region of equiatomic composition, the TiNi compound crystallizes in a cubic bcc lattice with the B_2_ structure. Within the Ti-Ni-Nb isothermal triangle, the formation of the ternary compounds TixNiyNbz with limited areas of homogeneity is observed (Figure 7). This phenomenon indicates a tendency to release intermetallic compounds of complex composition. It can be seen that the compound closest to the homogeneity region of the TiNi compound is the Ni_56_Ti_29_Nb_15_ compound (Figure 7, region 8). This is in good agreement with the fact that when TiNi based alloys are alloyed with Nb, the detected particles in the alloys studied have chemical compositions close to the composition of the compound.

In order to obtain precise information on the microstructure and phase composition of the material, thin foils were prepared for TEM. The technique of sample preparation included ionic thinning of the internal volumes of the material. The results of the structural analysis are shown in Figure 8. The corresponding microdiffraction patterns show reflections from the matrix B2 (TiNi) phase characterized by a volume-centered cubic lattice (Figure 8a). In addition to the main matrix phase, the most frequently encountered particles of Ti_2_Ni second phases are present in the samples (Figure 8c). The samples contain martensite lamellae with several orientational variants belonging to the B19′ phase (Figure 8b).

Ti_50_Ni_48.7_Nb_1_Mo_0.3_ alloy. Based on the analysis of quantitative microanalysis data, it was found that in the Ti_50_Ni_48.7_Nb_1_Mo_0.3_ alloy (Figure 9), the volume fraction of secondary phases decreases and particles of the Ni_56_Ti_29_Nb_15_ phase crystallize only along the grain boundaries (Figure 9). It was also found that the introduction of 1 at% Nb leads to a decrease in the amount of eutectic in the alloy. The average grain size in the alloy of this composition is 12 μm. Quantitative elemental analysis indicates the dissolution of Nb particles (0.28–0.45 at%) in the matrix and the precipitation of a phase with the composition Ni_56_Ti_29_Nb_15_ (Table 2).

Ti_50_Ni_48.2_Nb_1.5_Mo_0.3_ alloy. Analysis of micrographs of Ti_50_Ni_48.2_Nb_1.5_Mo_0.3_ alloys made it possible to establish that the alloy consists of a solid solution with areas of dendritic crystallization (Figure 10). In this alloy, during crystallization, a microstructural state without eutectic is formed, in contrast to alloys with 0.5 and 1 at%. The data of quantitative microanalysis are presented in Table 3. The Ti_50_Ni_48.2_Nb_1.5_Mo_0.3_ alloy consists of a TiNi solid solution with a small amount of Nb and Mo dissolved in it and areas of dendritic crystallization. Particles of the Ni_56_Ti_29_Nb_15_ phase crystallize along the boundaries of dendritic cells. In the alloy of this composition, a high content of Ti_2_Ni particles is observed, which is confirmed by the data of X-ray diffraction analysis.

It has been established that the average grain size in alloys with an increase in the Nb concentration to 1 at% decreases from 15 to 12 µm. This fact can be related to the fact that in these alloys, as was shown above, particles of the Ni_56_Ti_29_Nb_15_ compound are formed, which are located mainly along the grain boundaries and have an obstructive effect on the development and growth of grains. Thus, in alloys based on titanium nickelide of various compositions, similar structures are formed with features of distribution density, size, secondary phases, and their accumulations. A more uniform structure is observed in the Ti_50_Ni_48.7_Nb_1_Mo_0.3_ alloy with a predominant distribution of particles based on niobium along the grain boundaries.

### 4.3. Temperature Dependences of Electrical Resistance in Nb-Doped TiNi-Based Alloys

**Figure 11:**
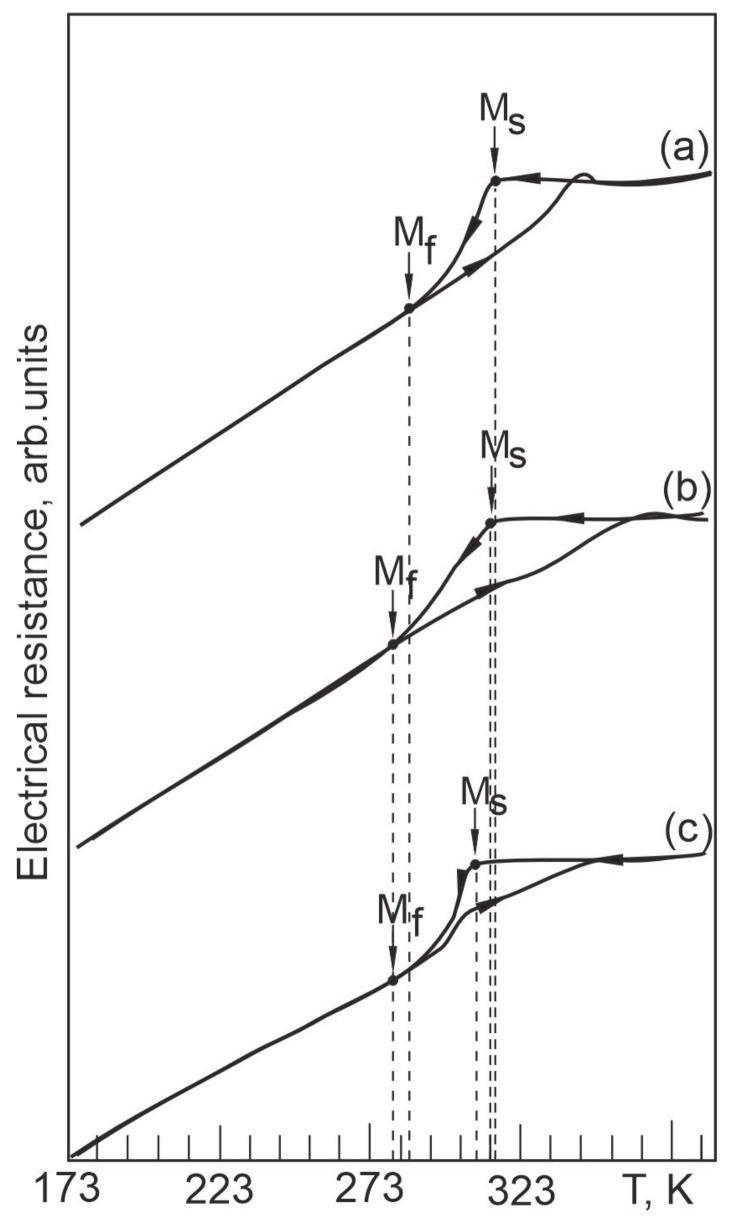
Dependences of electrical resistance ρ(t) on temperature in Ti_50_Ni_49.2_Nb_0.5_Mo_0.3_ (**a**), Ti_50_Ni_48.7_Nb_1_Mo_0.3_ (**b**), Ti_50_Ni_48.2_Nb_1.5_Mo_0.3_ (**c**) alloys.

To determine the temperature ranges of the MT during cooling/heating in the free state in TiNi-based alloys doped with Nb, the temperature dependences of the electrical resistance were obtained (Figure 11). The nature of the change in ρ(T) indicates that a single-stage MT occurs in the alloys studied from the initial B2 phase to the B19′ martensitic phase. Previously using X-ray diffraction analysis, it was found that the matrices of the studied alloys responsible for the thermoelastic martensitic transformations are in a two-phase state: austenitic B2 phase and martensite phase B19′ (see Section 4.1). According to the literature data on ternary TiNi(Nb) alloys [10,15,25] and our X-ray diffraction data, it can be concluded that the B2→B19′ MT occurs in these alloys.

**Figure 12:**
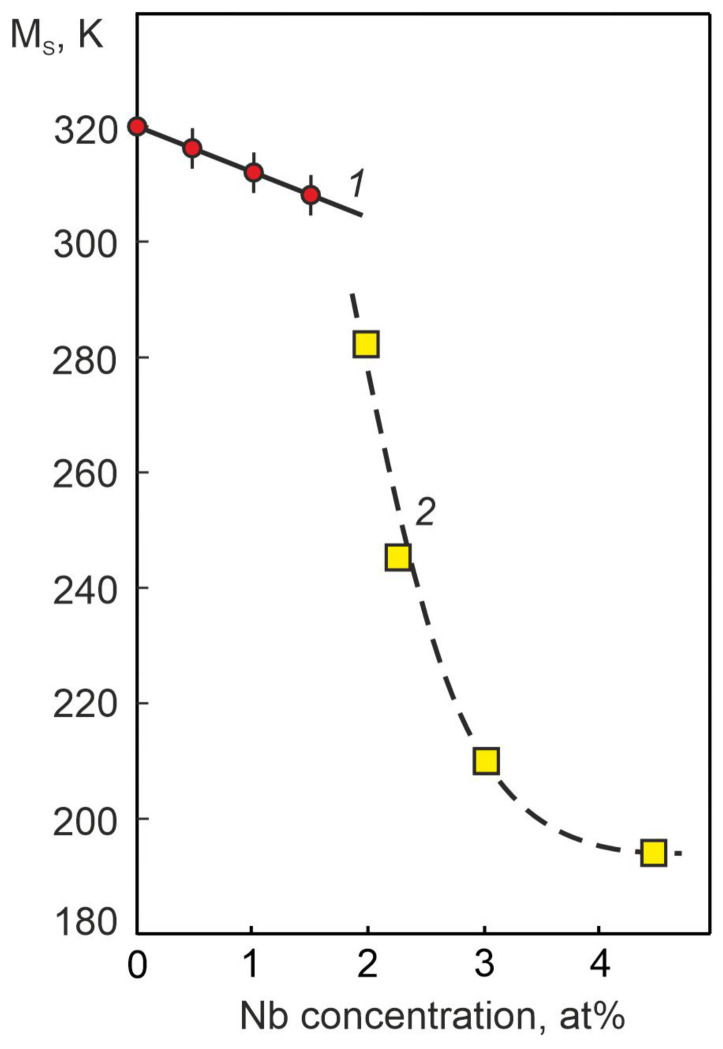
Dependences of temperature M_S_ on Nb concentration in alloys doped with Nb: (1)—Ti_50_Ni_49.7−X_Nb_X_Mo_0.3_ alloys according to our experimental data; (2)—Ti_50−X_Ni_50_Nb_X_ alloys according to the literature data [4,10,15].

An analysis of the obtained data showed that with an increase in the Nb concentration, the MT shifts to the region of low temperatures. Based on these temperature dependences, the concentration dependence of the temperature of the beginning of the forward MT M_S_ was obtained (Figure 12). It can be seen that alloying TiNi-based alloys with niobium according to the Ti_50_Ni_50−X_Nb_X_ scheme leads to a noticeable decrease in the M_S_ temperature.

In the literature, there are no works devoted to the study of the effect of low concentrations of Nb atoms during alloying of alloys based on titanium nickelide according to the Ti_50_Ni_50−X_Nb_X_ scheme on structure and phase composition. However, there are a number of works devoted to the study of the effect of doping with Nb atoms from 2 to 20 at% Nb of titanium nickelide according to the Ti_50−X_Ni_50_Nb_X_ and Ti_50−Y_Ni_50−X_Nb_Y+X_ schemes on the structural-phase states in wide temperature ranges [3,4,5,6,10,11,12,15,26,27,28]. Based on the analysis of literature data, the temperature dependence of the electrical resistance for TiNi-based alloys doped with Nb according to the Ti_50−X_Ni_50_Nb_X_ scheme was plotted (Figure 12, curve 2). A more intense decrease in the temperature of the onset of thermoelastic MT M_S_ is observed than in alloys based on TiNi alloyed with Nb according to the scheme Ti_50_Ni_50−x_Nb_X_. These data correlate with the results obtained in [29], where it was found that Nb atoms prefer to occupy titanium positions on the crystal lattice sites in TiNi and Ti_2_Ni intermetallic compounds rather than nickel positions. This tendency contributes to the stability of the B2 phase in TiNi (Nb) alloys doped according to the Ti_50−X_Ni_50_Nb_X_ scheme and, consequently, to a more intense decrease in the MT temperature with an increase in the concentration of the alloying component.

### 4.4. Shape Memory Effect in Nb-Doped TiNi-Based Alloys

**Figure 13:**
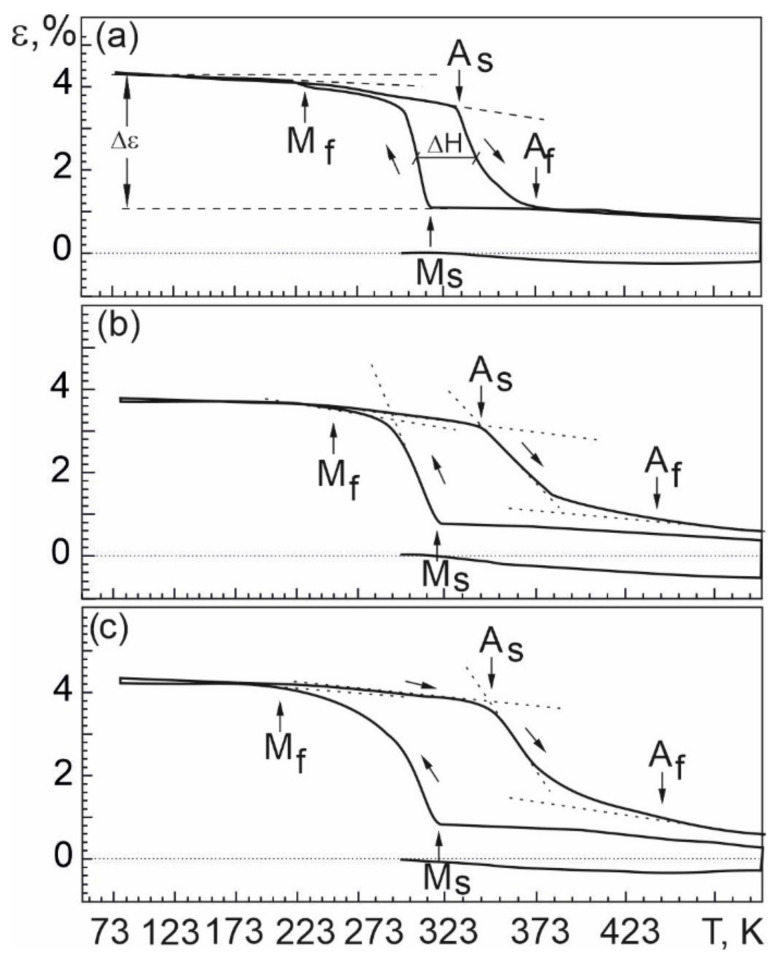
Multiple SME in alloys: Ti_50_Ni_49.2_Nb_0.5_Mo_0.3_ (**a**), Ti_50_Ni_48.7_Nb_1_Mo_0.3_ (**b**), Ti_50_Ni_48.2_Nb_1.5_Mo_0.3_ (**c**).

As a result of experimental studies, the temperature dependences of the accumulation and recovery of deformation under multiple shape memory effects in the alloys were obtained (Figure 13). From these ε(T) dependences, the characteristic temperatures of the MT (M_S_, M_f_, A_S_, A_f_) and the temperature MT intervals under load were determined [1,2].

Using the thermodynamic description of thermoelastic MTs and the experimental values of the characteristic MT temperatures obtained on samples under load and without load, we evaluated how Nb doping affects the ratio of the reversible $\left|\Delta \right.\left.G_{rev}(1)\right|$ and irreversible $\left|\Delta \right.\left.G_{fr}\right|$ components of the nonchemical free energy. The thermodynamic analysis of the MT features without load (in free state) and under load was carried out based on the analysis of the temperature interval $A_{f}-M_{s}$ in TiNi-based with Nb. This is due to the fact that, according to Equation (7), the value of the temperature interval $A_{f}-M_{s}$ depends on the Gibbs irreversible free energy ${\Delta G}_{fr}$.

**Figure 14:**
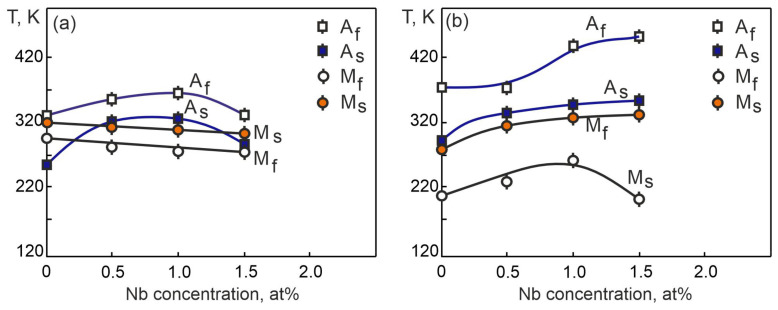
Dependences of the MT characteristic temperature based on the temperature dependences of the electrical resistance curves (**a**) and mechanical ε(T) tests (**b**).

Figure 14a shows the concentration dependences of the MT characteristic temperatures, obtained on the basis of the ρ(T) given in Figure 11. It can be seen that the change of the characteristic temperatures of the direct MT (M_s_ and M_f_) and the reverse MT ($A_{f}$ and $A_{s}$) on the concentration of the alloying component Nb have different functional dependences. This reflects the different influence of the concentration of the alloying element on the mobility of the interfacial boundaries in the case of forward and reverse MT. This is confirmed by the concentration curves of the difference in the characteristic temperatures of the MT $A_{f}-M_{s}$, shown in Figure 14a. The Nb doping of the titanium nickelide up to 1 at% leads to a linear increase in the temperature interval $A_{f}-M_{s}$. Further doping with Nb atoms leads to a decrease in the temperature interval $A_{f}-M_{s}$. Such a change in the temperature range $A_{f}-M_{s}$ is due to structural changes in the alloy: a significant increase in the lattice parameter a_B2_ of the matrix phase based on titanium nickelide with the B2 structure (Figure 5), due to a higher dissolution of Nb atoms in the solid solution (Table 3), as well as a higher content of the Ti_2_N phase particles. The value of the temperature interval $A_{f}-M_{s}$, according to Equation (7), depends on the Gibbs irreversible free energy ${\Delta G}_{fr}$. Thus, a decrease in the temperature range $A_{f}-M_{s}$ in the Ti_50_Ni_48.2_Nb_1.5_Mo_0.3_ alloy leads to a decrease in the irreversible Gibbs free energy ${\Delta G}_{fr}$.

Figure 14b shows an analysis of the concentration dependences of the characteristic MT temperatures under the load, obtained on the basis of the ε(T) given in Figure 13. With an increase in the Nb concentration in titanium nickelide alloy, the characteristic MT temperatures under the load M_S_, A_S_, A_f_ increase, while the M_f_ temperature changes monotonically with an increase in the concentration of Nb atoms. At the same time, with an increase in the concentration of niobium, an increase in the interval of manifestation of the SME is observed due to the difference between the temperatures A_f_ − M_f_. It is possible that Ti_2_Ni and Ni_56_Ti_29_Nb_15_ particles make an additional contribution to the expansion of the MT temperature range, preventing the completion of the forward and reverse MT by reducing the temperatures M_f_ and increasing A_f_ (Figure 14). Different functional dependences of the onset temperatures of the direct MT M_S_ in TiNi (Nb,Mo) alloy samples in the free state and under the load are probably due to particles precipitated along the grain boundaries. The presence of these particles along the grain boundaries, as the temperature of the alloy decreases under load, promotes the transition and facilitates the formation of martensite crystals. During cooling in the unstressed state, the grain boundary particles are an obstacle to the onset of MT.

**Figure 15:**
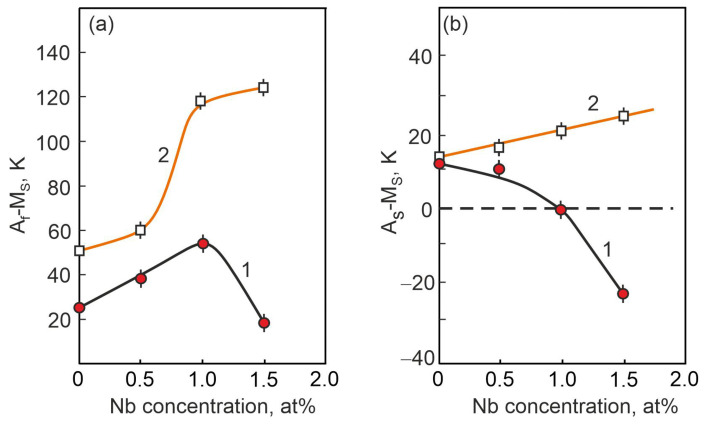
Dependences of the difference between the characteristic temperatures of MT A_f_ − M_s_ (**a**) and A_s_ − M_s_ (**b**) on the Nb concentration in alloys obtained from the temperature dependences of the electrical resistance curves (curve 1) and mechanical ε(T) tests (curve 2).

Further analysis of the features of the MT flow in samples without load and under load was carried out on the basis of the above thermodynamic relations (1)–(9). There is a completely different dependence of the thermal hysteresis A_f_ − M_s_ of the MT on the concentration of the Nb in TiNi-based alloys, determined when studying the MT under load (Figure 15, curve 2), compared to a similar dependence obtained when studying the MT in the free state (Figure 15, curve 1). In Figure 15 (curve 2), the temperature interval A_f_ − M_s_ increases stepwise with an increase in the concentration of Nb atoms from 0.5 at% Nb to 1 at%. Such a change in the temperature interval A_f_ − M_s_ with concentration reflects a sharp increase in the irreversible Gibbs free energy ΔG_fr_ (according to Equation (7)).

According to expression (7), the values of A_f_ − M_s_ depend on the irreversible non-chemical free energy $\left|\Delta \right.\left.G_{fr}\right|$ which is responsible for the irreversible dissipation of energy during the MT. The width of the thermal hysteresis A_f_ − M_s_ of the MT in alloy samples with an alloying element concentration of up to 0.5 at% Nb is slightly greater under load than in the same alloys without load. This indicates that in alloys under load during MT, the irreversible component of the non-chemical free energy $\left|\Delta \right.\left.G_{fr}\right|$ is higher than in alloys without load. In alloys with higher concentrations of the alloying element up to 1.5 at% Nb, a significant increase in irreversible non-chemical energy $\left|\Delta \right.\left.G_{fr}\right|$ is observed. Whereas in this significantly same alloy with an alloying element concentration of 1.5 at% Nb, the MT flow without load and the energy dissipation during the MT are less (Figure 15a). According to the data of [20], the main contribution to the energy dissipation $\left|\Delta \right.\left.G_{fr}\right|$ is made by the work on overcoming the stresses of resistance to the motion of the interfacial boundary. This work depends on the value of the Peierls energy barrier in the initial matrix and on the forces of interaction of interfacial boundaries with various defects (interstitial atoms, dislocations, dispersed particles) contained in the initial phase. Thus, in the Ti_50_Ni_48.2_Nb_1.5_Mo_0.3_ alloy, the precipitation of Ni_56_Ti_29_Nb_15_ particles, both along the grain boundaries and in the grain body, has no additional effect on the dissipation energy $\left|\Delta \right.\left.G_{fr}\right|$ during the motion of interfacial boundaries during MT. Whereas in the same alloy with the MT flow under load, the presence of precipitated particles leads to additional energy dissipation and is reflected by an increase in the values of $\left|\Delta \right.\left.G_{fr}\right|$.

A further search of MT features in the alloys was carried out on the basis of an analysis of the well-experimentally determined A_S_ − M_S_ temperature range, which depends on the difference between the irreversible Gibbs free energy $\left|\Delta \right.\left.G_{fr}\right|$ and the reversible one $\left|\Delta \right.\left.G_{rev}\right|$, according to Equation (8). The concentration dependence of the A_S_ − M_S_ MT temperature range flowing without load (Figure 15b, curve 1) shows a negative value of the A_S_ − M_S_ temperature difference for the Ti_50_Ni_48.2_Nb_1.5_Mo_0.3_. alloy. This indicates that the reversible Gibbs free energy ΔG_rev_ is more than twice as large as the irreversible Gibbs free energy ΔG_fr_. This phenomenon correlates with a decrease in the Gibbs irreversible free energy ΔG_fr_, which manifests itself in a decrease in the temperature interval A_f_ − M_s_ in the Ti_50_Ni_48.2_Nb_1.5_Mo_0.3_ alloy (Figure 15a, curve 1). In the same alloy, a completely different concentration dependence of the A_S_ − M_S_ MT temperature range under load is observed (Figure 15b, curve 2), which was obtained from the analysis of the ε(T) dependences. It can be seen that the A_S_ − M_S_ temperature range increases linearly with increasing Nb concentration in titanium nickelide. And as a result, in the Nb-doped TiNi alloy, martensitic transformation under load proceeds under different conditions, when the irreversible Gibbs free energy $\left|\Delta \right.\left.G_{fr}\right|$ is more than twice the reversible Gibbs free energy $\left|\Delta \right.\left.G_{fr}\right|$, according to Equation (8).

Thus, in the Ti_50_Ni_48.2_Nb_1.5_Mo_0.3_ alloy, in addition to the precipitation of dispersed Ni_56_Ti_29_Nb_15_ particles, both along the grain boundaries and in the grain body, a significant increase in the lattice parameter and atomic volume of the B2 phase is observed (Figure 5). This phenomenon reflects a more significant dissolution of the alloying element atoms in the B2 matrix phase based on the TiNi alloy. Such an additional dissolution of Nb atoms in the B2 matrix phase leads to a change in the forces of interatomic interaction. This manifests itself in a decrease in the temperature of the onset of MT M_S_ (Figure 12, curve 1) and a significant increase in the intensity of the (110)_B2_ reflection on the X-ray pattern (Figure 4 and Figure 5). In our case, the established structural changes reflect an increase in the stability of the B2 austenite phase. As a consequence, the increase in the stability of the austenite phase B2 correlates with the increase in the irreversible Gibbs free energy ΔG_fr_ during the MT flow under load, reflecting that a higher value of the MT driving force is required.

**Figure 16:**
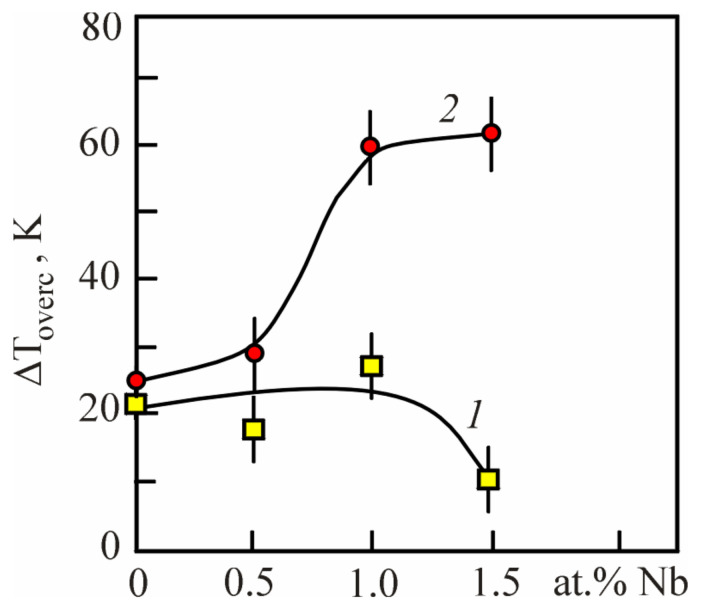
Dependences of the overcooling value ΔToverc of the alloy in the MT region relative to To on the concentration of Nb in alloys, obtained from the temperature dependences of the electrical resistance curves (curve 1) and from the dependencies ε(T) under load (curve 2).

Accordingly, a decrease in the temperature Ms reflects an increase in the stabilization of the B2 matrix phase and is accompanied by a change in the lattice parameters of the phases [30,31]. As noted earlier, an important fact here is the correlation in the change in the lattice parameter of the B2 phase depending on the Nb concentration in the three-component TiNi(Nb) alloys (Figure 5). This phenomenon is associated with the magnitude of overcooling of the alloy relative to the temperature T_0_ at which the chemical Gibbs energy of the austenitic and martensitic phases is the same. Based on Equations (1) and (2), we can estimate the value of the temperature interval of alloy overcooling, which depends on external mechanical stresses [20]. Such an assessment was performed and the results are presented in Figure 16. It can be clearly seen that for the MT to proceed under load, in alloys with an alloying element concentration of more than 1.0 at% Nb, more significant overcooling is necessary. Whereas for the MT to proceed without load, such a value of overcooling in the same alloys is not required. These data correlate with the conclusions obtained from the results of the analysis of the concentration dependences of the A_S_ − M_S_ temperature range (Figure 15b).

## 5. Conclusions

1. Structural studies showed that all alloys at room temperature consist of intermetallic compounds with the TiNi (B2, B19′), Ti_2_Ni, and Ni_56_Ti_29_Nb_15_ compositions. It was found that in the Ti_50_Ni_48.2_Nb_1.5_Mo_0.3_ alloy, a microstructural state without eutectic is formed during crystallization, in contrast to alloys with 0.5 and 1 at% Nb. It was established that the doping of TiNi alloys with niobium leads to an increase in the stability of high-temperature B2 phase. An increase in the lattice parameter and atomic volume of the B2 phase indicates that some of the Nb atoms are dissolved in the B2 matrix phase. The observed significant change in the structural-phase state has a significant effect on the features of the martensitic transition in the free state and under load.
2. In order to determine the characteristics of martensitic transformation in the free state and under load in TiNiNb based alloys, temperature dependences of electrical resistivity and temperature dependences of strain accumulation and recovery under multiple shape memory effects were obtained. The dependencies indicate that a single stage B2–B19′ MT occurs in the alloys studied. From the experimental data, characteristic temperatures and intervals of MT were determined. Using the thermodynamic description of thermoelastic MT and the experimental values of the characteristic temperatures based on the dependences obtained in the free state and under load, the influence of Nb alloying on the ratio of the reversible and irreversible components of the non-chemical free energy was evaluated. These studies play an important role in determining the behavior of the alloys under different operating conditions, namely in the free state and under load.
3. The width of the thermal hysteresis A_f_ − M_s_, corresponding to the irreversible energy dissipation during MT, was determined. In the experiments under load, it was found that the alloys show a significant increase in the irreversible free energy dissipation during MT, whereas in the free state the energy dissipation during MT is much lower in the same alloys. This indicates that the irreversible component of the non-chemical free energy is higher in the loaded alloys during MT than in the unloaded alloys, and that particle precipitation has an additional influence on the dissipation energy during interfacial boundary motion under load.
4. The temperature difference A_S_ − M_S_ between the beginning of the direct and the beginning of the reverse MT, determined by the ratio between the reversible and irreversible energies, was established. Based on the analysis of the concentration dependence of the temperature interval A_S_ − M_S_ during MT under load, it was found that alloying with Nb causes the reversible Gibbs free energy $\left|\Delta \right.\left.G_{rev}\right|$ to be more than twice as large as the irreversible Gibbs free energy $\left|\Delta \right.\left.G_{fr}\right|$. In the same alloy, a completely different concentration dependence of the temperature interval A_S_ − M_S_ MT occurring under load is observed when the irreversible Gibbs free energy exceeds more than twice the reversible Gibbs free energy.
5. The MT mechanism strongly depends on the concentration of the alloying element, and this is reflected in the change in the ratio between the reversible $\left|\Delta \right.\left.G_{rev}\right|$ and irreversible $\left|\Delta \right.\left.G_{fr}\right|$ Gibbs free energies at MT flow without load and under load. These changes in the concentration dependences of the MT temperature intervals in the alloys studied in the free state and under load depend on the precipitation of particles along the grain boundaries and in the grain body and on the changes in lattice parameter and atomic volume in the austenitic B2 phase.
6. Based on the analysis of the concentration dependences of the temperature T_0_ of the chemical equilibrium of the austenitic and martensitic phases, it was found that for the MT to occur under load, in alloys with an alloying element concentration of more than 1.0 at% Nb, a more significant overcooling is required than for MT without load.

## Figures and Tables

**Table 1:** Quantitative elemental composition of the structural components of the Ti_50_Ni_49.2_Nb_0.5_Mo_0.3_ alloy.

Structural Component	Composition, at%			
	Ti	Ni	Mo	Nb
*B2* matrix	41.3 ± 0.5	58.3 ± 0.5	0.14 ± 0.1	0.3 ± 0.5
Ti_2_Ni particles	65.4 ± 0.5	34.6 ± 0.5	–	–
Ni_56_Ti_29_Nb_15_ particles	29.8 ± 0.5	55.9 ± 0.5	–	14.3 ± 0.5

**Table 2:** Quantitative elemental composition of the structural components of the Ti_50_Ni_48.7_Nb_1_Mo_0.3_.

Structural Component	Composition, at%			
	Ti	Ni	Mo	Nb
*B2* matrix	40.1 ± 0.5	59.2 ± 0.5	0.2 ± 0.1	0.4 ± 0.1
Ti_2_Ni particles	63.3 ± 0.5	36.7 ± 0.5	–	–
Ni_56_Ti_29_Nb_15_ particles	30.3 ± 0.5	53.6 ± 0.5	–	16.1 ± 0.5

**Table 3:** Quantitative elemental composition of the structural components of the Ti_50_Ni_48.2_Nb_1.5_Mo_0.3_.

Structural Component	Composition, at%			
	Ti	Ni	Mo	Nb
*B2* matrix	38.0 ± 0.5	61.2 ± 0.5	0.3 ± 0.1	0.5 ± 0.1
Ti_2_Ni particles	61.5 ± 0. 5	38.6 ± 0.5	–	–
Ni_56_Ti_29_Nb_15_ particles	29.3 ± 0.5	54.8 ± 0.5	–	16.0 ± 0.1

## Data Availability

Data are contained within the article.
